# High-Resolution Chloroplast SNV Profiling of 409 Grapevine (*Vitis vinifera* L.) Cultivars Using Whole-Genome Shotgun Sequencing

**DOI:** 10.3390/ijms27031583

**Published:** 2026-02-05

**Authors:** Katarina Rudolf Pilih, Tomaž Kasunič, Tjaša Cesar, Denis Rusjan, Mitra Razi, Tatjana Jovanović-Cvetković, Aida Dervishi, Dragoslav Ivanišević, Katerina Biniari, Klime Beleski, Vesna Maraš, Goran Zdunić, Ana Mandić, Roberto Bacilieri, Jernej Jakše, Nataša Štajner

**Affiliations:** 1 Biotechnical Faculty, University of Ljubljana, 1000 Ljubljana, Slovenia; katarina.rudolf.pilih@bf.uni-lj.si (K.R.P.); tomaz.kasunic@jafral.com (T.K.); tjasa.cesar@bf.uni-lj.si (T.C.); denis.rusjan@bf.uni-lj.si (D.R.); mitra.razi@bf.uni-lj.si (M.R.); 2 Jafral d.o.o., Stegne 13A, 1000 Ljubljana, Slovenia; 3 Faculty of Agriculture, University of Banjaluka, Bulevar Vojvode Petra Bojovića 1A, 78000 Banja Luka, Bosnia and Herzegovina; tatjana.jovanovic-cvetkovic@agro.unibl.org; 4 Department of Biotechnology, Faculty of Natural Sciences, University of Tirana, Sheshi Nene Tereza 4, 1010 Tirane, Albania; aida.dervishi@fshn.edu.al; 5 Faculty of Agriculture, University of Novi Sad, Trg. D. Obradovića 8, 21000 Novi Sad, Serbia; dragoslav@polj.uns.ac.rs; 6 Faculty of Crop Science, Agricultural University of Athens, 75 Iera Odos, 11855 Athens, Greece; kbiniari@aua.gr; 7 Institute of Agriculture, Ss. Cyril and Methodius University, Ulica 16ta Makedonska Brigada 3A, 1000 Skopje, North Macedonia; k.beleski@zeminst.edu.mk; 8 Biotechnical Faculty, University of Montenegro, Mihaila Lalića Br. 15, 81000 Podgorica, Montenegro; vesna.maras@ucg.ac.me; 9 Institute for Adriatic Crops and Karst Reclamation, Put Duilova 11, 21000 Split, Croatia; goran.zdunic@krs.hr; 10 Centre of Excellence for Biodiversity and Molecular Plant Breeding, Svetošimunska cesta 25, 10000 Zagreb, Croatia; 11 Faculty of Agriculture and Food Technology, University of Mostar, Biskupa Čule bb, 88000 Mostar, Bosnia and Herzegovina; ana.mandic@aptf.sum.ba; 12 UMR 1334 AGAP, INRAE/CIRAD, Bâtiment 3, Bureau 103, TA A-108/03, Avenue Agropolis, 34398 Montpellier Cedex 5, France

**Keywords:** chloroplast genome, grape DNA, Ion Torrent, molecular phylogeny, SNV

## Abstract

The grapevine (*Vitis vinifera* L.) is one of the most important horticultural crops, with thousands of varieties cultivated worldwide. In this study, we analyzed chloroplast SNV markers using a whole-genome shotgun sequencing approach to investigate the genetic diversity and phylogeny of 409 cultivated *V. vinifera* accessions originating from nine countries across Southeast and Central Europe, as well as a heterogeneous set of additional accessions maintained by INRAE. Shotgun sequencing allowed high coverage, enabling the detection of 93 SNVs across 24 chloroplast genes, including 11 non-synonymous variants. The *ycf1* gene showed the highest variability, consistent with its role in species differentiation. Haplotype analysis revealed 102 distinct haplotypes, with clear geographic structuring: ATT predominated in the eastern Mediterranean, ATA in western Europe, and GTA mainly in a heterogeneous group of varieties from a French collection. To validate the shotgun approach, seven SNV markers were analyzed using target capture sequencing, confirming the accuracy of detected variants with only minimal discrepancies, which is mostly attributable to homopolymeric regions and low-frequency alleles. Phylogenetic analyses using both trees and networks delineated three major haplotype clusters, reflecting human-mediated dispersal of grapevine cultivars through historical viticultural practices. This study represents the largest chloroplast genome analysis of cultivated *V. vinifera* to date, providing a large cpDNA resource for assessing chloroplast diversity and maternal haplotype structure in cultivated grapevine. The results highlight the power of combining high-throughput sequencing and chloroplast genomics for population-level studies in perennial crops.

## 1. Introduction

Grapevine (*Vitis vinifera* L.) is an important fruit crop widely cultivated around the world and valued for its high nutritional content [1,2]. It is estimated that approximately 8000 to 10,000 grape cultivars are grown worldwide [3]. The genus *Vitis* comprises about 70 species, most of which are native to Asia and North-Central America, with only one species occurring naturally in Europe [4,5,6]. Domestication of *V. vinifera* began 6000–8000 years ago in the Near East [7] and then the cultivars reached most European countries, North Africa and the East through different routes. Old cultivated grapevine is thought to have been domesticated from a wild population of *Vitis vinifera* ssp. *sylvestris* (C. C. Gmel.) Hegi [8]. There are strong morphological differences between the two subspecies. The subspecies *sylvestris* is dioecious with male and female flowers and prefers humid environments, while cultivated grapevines are monoecious with predominantly hermaphrodite flowers and are often accustomed to relatively dry environments [9]. Grapevine domestication was a complex and regionally structured process, involving multiple domestication centers, gene flow between wild and cultivated populations, and extensive vegetative propagation. This process resulted in important genetic changes, including the transition from dioecy to hermaphroditism, selection of larger and sweeter berries, variation in fruit color, and the establishment of clonal lineages [10]. By comparing multiple grapevine genomes, population genomics revealed both the acquisition of genes affecting fruit quality and the loss of genes involved in disease resistance and sexual reproduction, showing the limitations of chloroplast-based approaches in detecting these domestication-driven changes [11]. Nevertheless, chloroplast genome analyses provide valuable and complementary information on maternal lineages, population structure, and historical diffusion patterns, particularly when integrated with nuclear genome data.

Because of repeated interspecific hybridization and intricate morphological diversity, the classification of *Vitis* species is controversial [12]. In the last century, historical documents combined with ampelographic data have been used to investigate the origin and relationships between grape varieties. Integrative approaches combining molecular and morphological data have been shown to improve resolution of species relationships within *Vitis* [13].

Nowadays, with the development of molecular methods, it is possible to investigate the genetic relationships between grape cultivars and their relationship with wild genotypes [14]. Some DNA markers including RFLP (Restriction Fragment Length Polymorphism), RAPD (Random Amplified Polymorphic DNA), SSR (Simple Sequence Repeats), and SNV (Single Nucleotide Variant) have been utilized in genetic studies of grapevine [15,16,17]. SNVs are the most abundant type of sequence variations in plant genomes [18]. They are particularly useful for analyzing genetic diversity and for plant breeding programs that require numerous markers to achieve comprehensive genome coverage [19]. Next-generation sequencing (NGS) provides a high-throughput and cost-effective molecular tool for plant breeding and has been widely applied to accelerate breeding processes [20,21] using various NGS platforms. Among the major NGS platforms, the Ion Torrent system is noted for having one of the lowest instrument costs and is the first post-light sequencer [22,23]. Plastid genomics provides important context for grapevine domestication, revealing maternal lineages and haplotype diversity in wild and cultivated *Vitis vinifera* populations [24]. Chloroplast DNA (cpDNA), due to its conserved structure, gene content and slow evolutionary level, has a unique value in plant evolution [25], phylogeographic studies [26], taxonomy [27], and diversity evaluation [28]. Chloroplast SNVs are a novel type of molecular markers that exhibit the typical features of SNV markers within the chloroplast genome [29] and the research on the chloroplast genome has been reported to be very valuable in plant phylogenetic studies [30]. The chloroplast genome of grapes is 160,928 bp in length [31] containing large and small single-copy regions and a pair of identical inverted repeat sequences (IRs) [32]. With the development of high-throughput sequencing, access to the grape chloroplast genome has recently become more readily available [33]. Due to its high economic importance, it is understandable that within the genus *Vitis*, the chloroplast genome was first sequenced in *V. vinifera* [31]. Afterward, the chloroplast genomes of some other members of this genus, e.g., *V. berlandieri* [34], *V. pseudoreticulata* [32], *V. riparia* [6] and *V. amurensis* [35], were also sequenced and assembled de novo for the purposes of phylogenetics and breeding. SNV markers on cpDNA are well-suited for identifying phylogenetic relationships among grapevine varieties. For example, resequencing of the complete cpDNA of 11 Georgian varieties revealed a total of 86 SNVs [36]. Their results indicate that the cpDNA of several of these varieties is almost indistinguishable from that of Western European varieties. Several studies have also investigated the phylogeny of the genus *Vitis* through analyses of chloroplast SNVs [30,31,37,38,39]. While chloroplast SNPs provide insights into maternal lineages, they cannot detect introgression events, which have been documented in cultivated grapes through genome-wide analyses [40]. Recent nuclear genome studies have resolved aspects of grapevine domestication, clonal diversification, structural variation, and introgression, providing genome wide context beyond chloroplast markers [11,13,40,41,42,43]. In contrast, chloroplast SNVs primarily track maternal lineages and offer complementary resolution for lineage sharing among cultivars. Our study therefore focuses on cpSNV haplotyping in cultivated *V. vinifera* L. without attempting demographic reconstructions.

In this study, we analyzed chloroplast SNV markers using a whole-genome shotgun sequencing approach to investigate intraspecific genetic diversity and phylogenetic relationships within cultivated grapevine, *V. vinifera* L. The analysis was performed on 409 grapevine accessions (cultivars) originating from nine countries across Southeastern and Central Europe and complemented by a heterogeneous set of additional cultivated *V. vinifera* accessions maintained by INRAE and originating from Europe, the Caucasus, and Central Asia (Supplementary Table S1). Hereafter, “accessions” refers to individual grapevine genotypes or clonal entries maintained in institutional collections. Importantly, all analyzed accessions represent cultivated forms of *V. vinifera*, and wild grapevine (*V. vinifera* ssp. *sylvestris*) was not included in this study.

By restricting the analysis to cultivated *V. vinifera*, this study focuses on chloroplast genome variability and phylogenetic structure at the cultivar level. The chloroplast genome is used here as a complementary marker to explore maternal lineage patterns rather than to reconstruct complete phylogenetic relationships, allowing the assessment of maternal lineage diversity and broad geographic structuring within cultivated grapevine. To validate the shotgun-based results, seven SNV chloroplast markers obtained via target capture sequencing were additionally analyzed. This combined strategy enabled robust identification of chloroplast SNVs from whole-genome shotgun data, while targeted capture sequencing of selected loci provided additional validation of the detected variation.

## 2. Results

### 2.1. Sequencing and Mapping

The shotgun sequencing, i.e., whole-genome sequencing of 409 grapevine accessions performed on the Ion Proton™ system, yielded a total of 422.5 million single-end reads (mean 1,033,004 reads per accession; median 882,908; range 232,204–7,090,504) with an average read length of 160.8 ± 8.68 bp (median 162.4 bp; range 132.9–179.3 bp). All sequencing data were deposited in the SRA under BioProject Accession PRJNA1366753.

**Figure 1:**
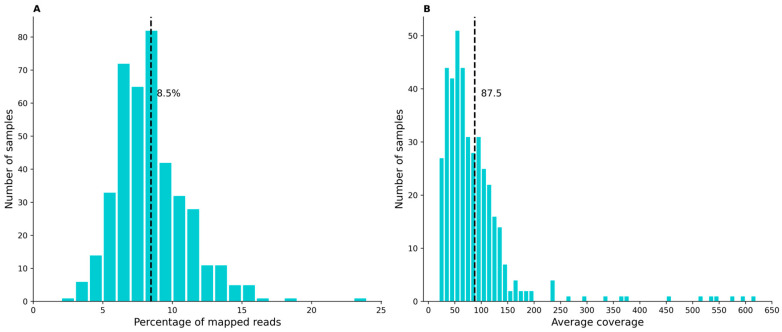
Percentage of reads mapped to the chloroplast genome (**A**) and average coverage of chloroplast genome (**B**) for 409 grapevine accessions.

After mapping the reads to the reference chloroplast genome (NC_007957), the average chloroplast genome coverage was 87.6× (standard deviation 77.7, range 21.0×–616.2×), reflecting substantial variability in chloroplast read depth across samples, as is expected for whole-genome shotgun sequencing. On average, 8.5% of all sequencing reads mapped to the chloroplast genome (standard deviation 2.6%, range 3.0–23.2%), consistent with differences in chloroplast DNA content among samples (Figure 1). Average mapped read length was 162.7 bp (standard deviation 8.7 bp, range 133.5–181.6 bp). All sequencing quality control metrics and mapping statistics are provided in Supplementary Table S2.

### 2.2. Detection of Chloroplast SNVs

**Figure 2:**
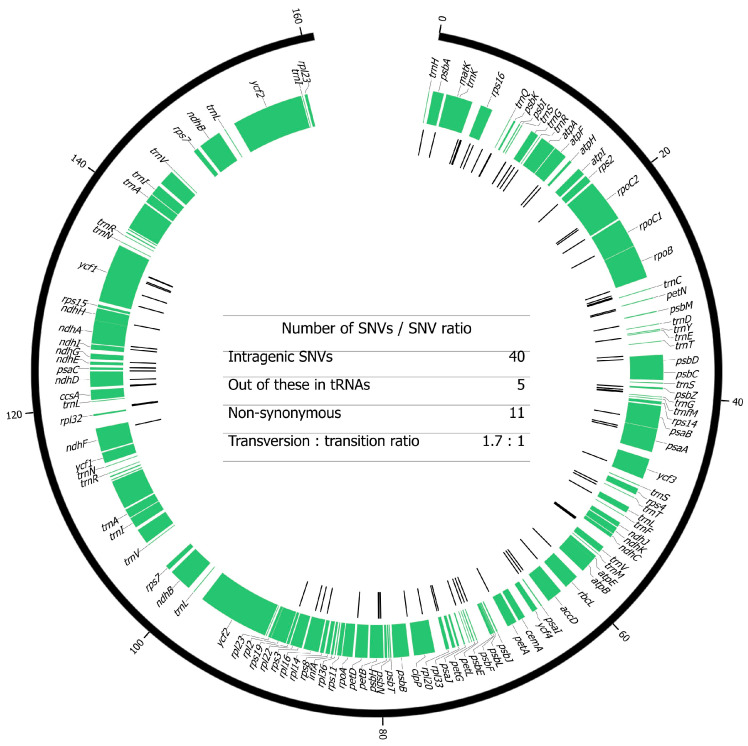
Single nucleotide variants (SNVs) in the gene map of the grapevine chloroplast genome. The light gray sections of the outer ideogram illustrate the positions of IRA and IRB, which were excluded from SNV calling. The green tracks represent the positions of the genes, with labeled names. The black lines indicate the positions of the SNVs found. In the middle of the figure is a table with types of the SNVs detected.

A total of 93 single nucleotide variants (SNVs) were detected in 409 chloroplast genomes (Supplementary Table S3 and Figure 2). The accessions SRB-14, HRV-132 and MKD-401 exhibited the highest number of SNVs, all sharing an identical set of 64 variants.

**Figure 3:**
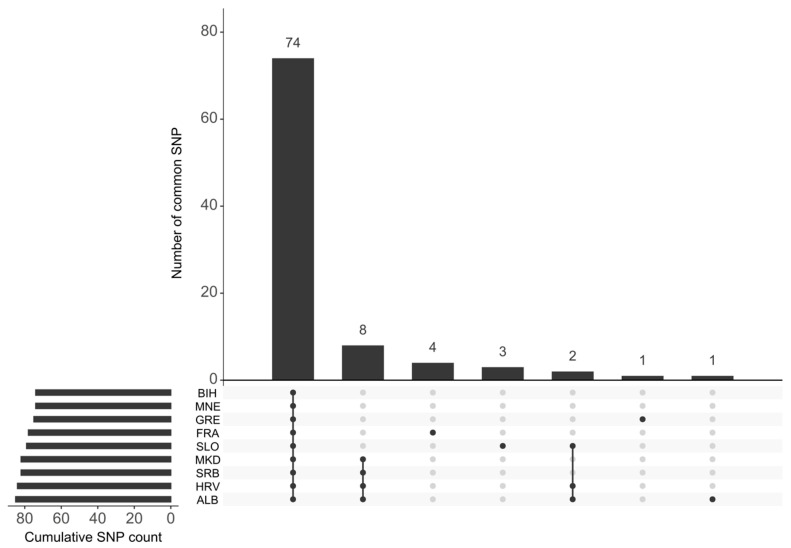
*UpSet* (v2.0) graph showing the number of detected SNVs by country of origin. The bar chart at the top of the graph indicates the total number of SNVs in each set intersection. Each intersection includes the clusters indicated by black dots connected by lines in the matrix below each column. The line chart in the lower left shows the cumulative number of detected SNVs by country. Country abbreviations are defined as follows: BIH (Bosnia and Herzegovina), MNE (Montenegro), GRE (Greece), FRA (France), SLO (Slovenia), MKD (North Macedonia), SRB (Serbia), HRV (Croatia), and ALB (Albania).

Two accessions, HRV-118 (‘Plavčina’) and BIH-609 (‘Alikant buše’), showed no detectable SNVs, indicating that their chloroplast genomes are identical to the reference sequence (‘Maxxa’). Among all accessions, 74 SNVs were shared across all datasets included in the study. Additionally, four SNVs were specific to the French collection dataset, and three SNVs were specific to the Slovenian varieties. Furthermore, a single SNV was found to be specific to the Greek and Albanian varieties, respectively (Figure 3).

### 2.3. SNV Analysis for Haplotyping

**Figure 4:**
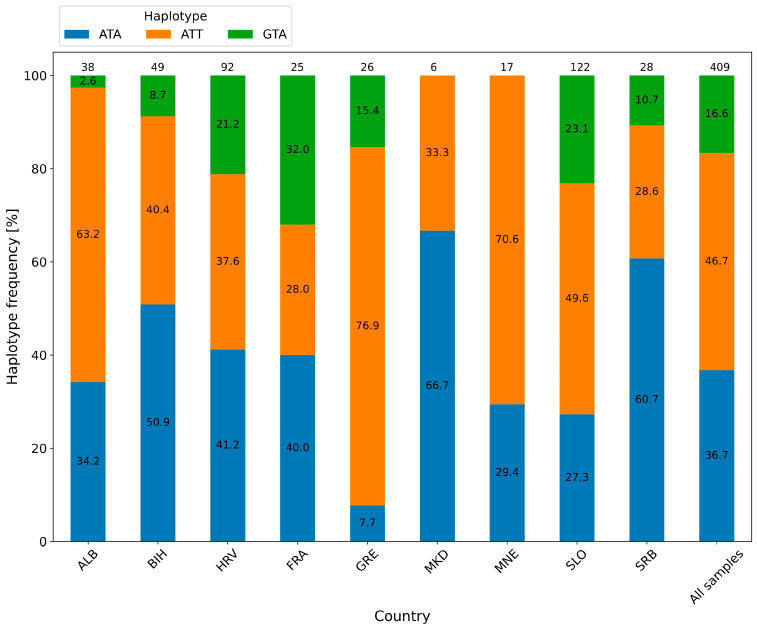
Bar chart showing the frequency of each haplotype according to the geographic origin of the grapevine accessions. The final bar represents the overall haplotype distribution across all 409 accessions. ALB: Albania; BIH: Bosnia and Herzegovina; FRA: France; GRE: Greece; HRV: Croatia; MKD: North Macedonia; MNE: Montenegro; SLO: Slovenia; SRB: Serbia.

To enable comparisons with previous studies [36,44,45], haplotypes were determined based on three chloroplast SNV sites (positions 205, 86715, and 86721). At site 86715, the SNV was not detected in any of the analyzed accessions. Using the other two sites, we identified three distinct haplotypes—ATA, ATT, and GTA—in a total of 409 accessions. Overall, the ATT haplotype was the most prevalent, present in 46.7% of the accessions, followed by ATA at 36.7% and GTA at 16.6% (Figure 4).

Using Fisher’s exact test, we confirmed that the distribution of haplotypes differs significantly according to the country of origin. The test performed on the complete 3 × 9 contingency table (Supplementary Table S4) revealed a statistically significant association between the variables (*p* = 0.000498), indicating that the null hypothesis of independence between haplotype distribution and geographic origin was rejected in favor of the alternative. Subsequently, we applied the same test to examine the associations for all possible haplotype–country combinations. The results of all 108 tests are reported as *p*-values in Supplementary Table S5. It was found that out of the 108 combinations, the association between variables was significant in nine cases (bolded in Table S5).

### 2.4. Phylogenetic Relationships and Haplotype Network of Grapevine Accessions

**Figure 5:**
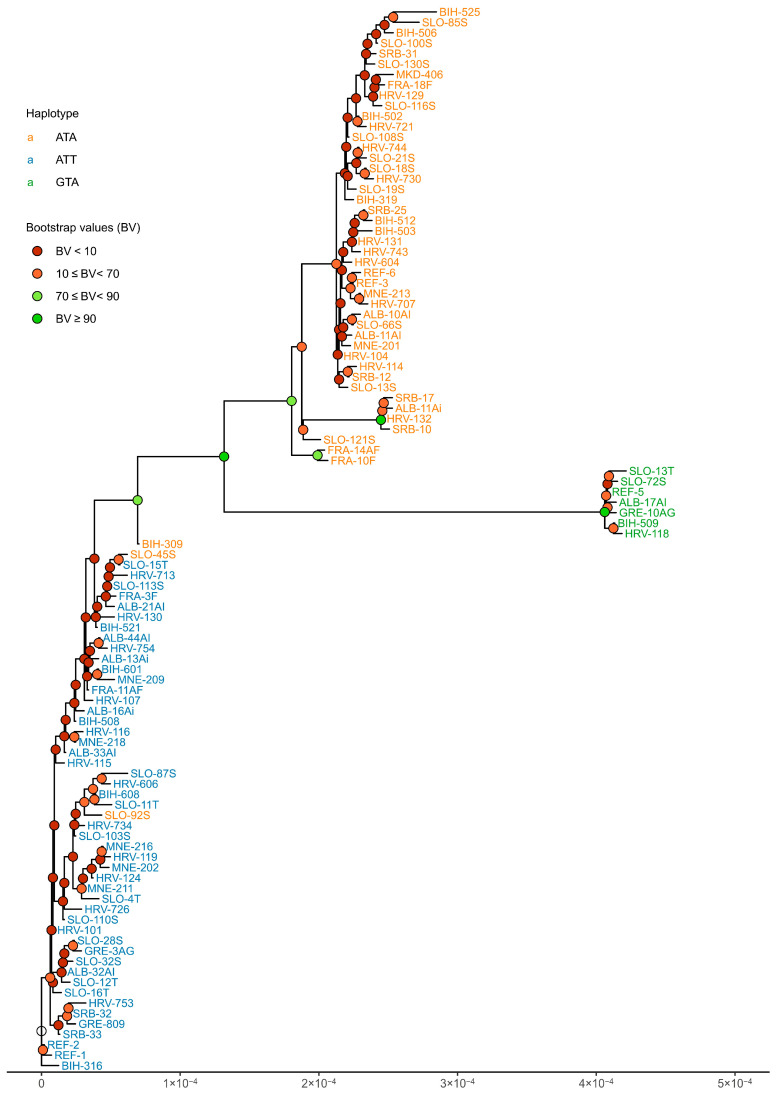
Phylogenetic tree of 102 unique alternative sequences obtained by maximum likelihood using RAxML tool (v8.0.0). Each node represents a single unique chloroplast sequence that may be shared by multiple grapevine accessions of different geographic origins. The node labels correspond to representative sample accessions, as defined in Supplementary Table S6, and do not imply that the geographic origin of all accessions share the same sequence. Accession labels are colored according to the haplotype of each alternative sequence. The internal nodes of the tree are colored according to the bootstrap values. The uncolored node corresponds to the tree root.

After removing duplicate alternative sequences, we obtained a set of 102 unique chloroplast sequences. Of these, 62 were specific to a single grapevine cultivar, while the remaining 40 were shared among the other 347 cultivars. The phylogenetic tree (Figure 5) revealed three clusters that largely correspond to the three defined haplotypes, with the exception of three accessions (SLO-45S, SLO-92S, and BIH-309).

**Figure 6:**
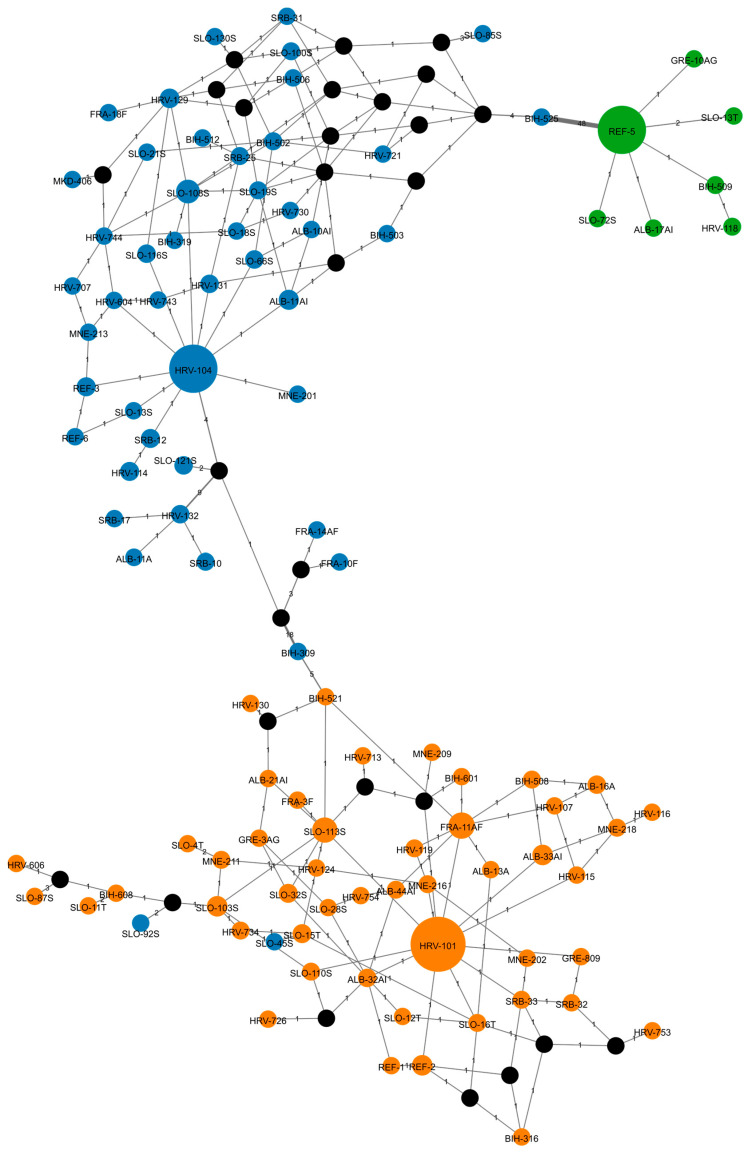
The phylogenetic network of the unique alternative grapevine sequences constructed using the Median-Joining (MJ) approach. Each node represents a single unique chloroplast sequence that may be shared by multiple grapevine accessions of different geographic origins. The node labels correspond to representative sample accessions, as defined in Supplementary Table S6, and do not imply that the geographic origin of all accessions share the same sequence. Nodes are colored according to the haplotype of each sequence. Node size reflects the frequency of the sequence in the complete accession set. Numbers alongside the connections and their thickness indicate the number of differences between neighboring nodes. Orange nodes represent ATA haplotype, green nodes GTA haplotype and blue nodes ATT haplotype. Black nodes represent Steiner points.

We further constructed a phylogenetic network of all 102 sequences using the Median-Joining (MJ) method implemented in PopART (Figure 6). The MJ algorithm introduced 28 Steiner points to the initial nodes. The network revealed three clearly separated clusters, demonstrating that the MJ method successfully grouped the alternative sequences according to the three defined haplotypes. As observed in the phylogenetic tree, accessions SLO-45S and SLO-92S cluster in a group that does not correspond to their assigned haplotype, whereas accession BIH-309 is correctly assigned in this analysis.

Among the 40 alternative reference sequences shared by multiple accessions (Supplementary Table S6), the most common sequence, labeled as HRV-101, corresponds to the ATA haplotype. This sequence was detected in 73 accessions and of these, 24 originated from Slovenia, corresponding to 19.7% of all Slovenian accessions included in the analysis. The next most frequent sequences are those labeled as HRV-104 and REF-5, each found in 60 accessions. Sequence HRV-104 is primarily represented by accessions from Croatia, including 15 of them, representing 17.6% of the Croatian dataset. The sequence labeled REF-5 is mostly represented by accessions from Slovenia, including 26 of them, representing 21.3% of the Slovenian dataset. The 62 accessions with unique sequences are listed in Supplementary Table S7, with the majority originating from Croatia (14) and Slovenia (14), and Bosnia and Herzegovina (12). It should be noted that the number of accessions differed substantially among countries, reflecting the size and availability of national and institutional germplasm collections. Therefore, the observed frequencies of shared alternative sequences are interpreted as representative of the analyzed collection rather than as estimates of population-level frequencies.

In the targeted approach, we used a set of seven hybridization probes specifically designed to capture and isolate previously characterized polymorphic regions of the chloroplast genome (Supplementary Table S8). This strategy enabled efficient enrichment of cpDNA fragments known to contain informative sequence variation. We detected all seven chloroplast SNVs, each present at varying frequencies across the 409 *V. vinifera* accessions, with alternative allele proportions ranging from less than 1% to nearly 47% (Table 1).

## 3. Discussion

Next-generation sequencing enables the simultaneous sequencing of millions of DNA molecules in a short time, providing high sequencing coverage [46] which is essential for the accurate detection of SNVs [47] and subsequent analyses of genetic diversity [48]. Chloroplast DNA (cpDNA) contains variable numbers of mononucleotide repeats that can be used to analyze phylogeographic patterns in plant species [30,49]. The chloroplast genome is highly conserved across *Vitis* species [50] and still contains sufficient genetic variation to identify the ancestral parents of grape cultivars. This study does not aim to reconstruct the domestication history of grapevine, but rather to describe chloroplast sequence diversity within cultivated germplasm. Because cpDNA is maternally inherited, it represents a small fraction of total cellular DNA, and exhibits lower polymorphism than the nuclear genome; cp-based trees/networks provide complementary but only partial views of grapevine history. Inferences about domestication processes, introgression, or clonal genealogy require nuclear data (WGS/SNP chips) and population genetic modeling; here, we interpret cpSNV clusters strictly as having maternal haplotype structures within conserved germplasm.

We aimed to obtain comprehensive chloroplast genome sequence data using a whole genome sequencing approach, followed by single nucleotide polymorphism analysis of the chloroplast genomes. As starting material, we used total cellular DNA extracted from young leaves. Total cellular DNA extracted from young leaves served as the starting material, as developing leaf tissue contains a high number of chloroplasts per cell [51]. Direct isolation of chloroplasts can be challenging in some species [52], and rapid isolation protocols often produce DNA templates that are heavily contaminated with nuclear DNA [53]. The successful sequencing of cpDNA from total cellular DNA of grapevine has been previously reported [33]. Using the same approach, we also achieved successful results, obtaining an average coverage of 88× for chloroplast genomes.

Using a shotgun approach, we detected 93 SNVs that are highly comparable to the variants discovered by Tabidze et al. [36] in the cpDNA of three Georgian varieties. Of the 86 SNVs detected in that study, 73 were in agreement with our results. This consistency is further reflected in the unexpectedly high proportion of transversions among the detected SNVs, with a transversion-to-transition ratio of 1.7. Since transitions involve less substantial chemical changes than transversions, the opposite trend would typically be expected. In nuclear genomes, transitions also generally outnumber transversions. The higher frequency of transitions is likely related to CpG dinucleotides, where methylated cytosines are more prone to deamination, leading to C-to-T transitions [54]. Regarding single nucleotide substitutions in cpDNA, the alga *Chlamydomonas reinhardtii* was shown to have a ratio shift towards transversions, supporting our results [55]. Among the detected SNVs, a total of 40 variants were found across 24 different genes. The highest number of variants (six) was found in the *ycf1* gene (Figure 2), which is frequently used for species differentiation due to its high variability and its large size; it can be used also for distinguishing closely related plant species [56]. Additionally, a total of five SNVs were identified in three different tRNA genes. Of the 35 SNVs located within protein-coding regions, 11 were non-synonymous, with the highest number (four) occurring in the *ycf1* gene.

Targeted capture sequencing of seven SNVs was performed to validate the accuracy of the shotgun approach for detecting chloroplast SNVs. Target sequencing has been used in many other plant species for phylogenetic studies of large populations. For example, in sugarcane, this procedure has been used to clarify the origin of modern varieties [57] and in oilseed rape, target sequencing has applied as a procedure for discovering new SNV markers [58]. The set of 24 probes was primarily designed for interspecific analysis of chloroplast SNVs in *Vitis* species [59], but in the present work we only used seven probes that were suitable for *V. vinifera* analysis. Comparison of detected variants between the targeted and shotgun approaches resulted in only 20 discrepancies out of all accession-locus (409 × 7) comparisons (Supplementary Table S9). Two sites, 73765 and 75398, proved to be perfect matches with the shotgun approach. One mismatch between the two approaches was found at sites 123690 and 128420. At site 123690, the polymorphism in accession BIH-309 was detected exclusively using the shotgun approach. SNV was not detected at this site in the targeted approach because the frequency of the alternative allele was low and the defined limit (35%) was not exceeded. At site 128420, the SNV of the ALB-2AI accession was also detected only in the shotgun approach. When reviewing the mapped reads obtained by the target approach, we observed that the ALB-2AI accession site was not covered by reads at all, indicating that the capturing of this locus was unsuccessful for this accession. Interestingly, however, the coverage of the chloroplast genome by shotgun approach was the highest for this accession. Two mismatches were detected at site 123664. In accessions BIH-309 and BIH-519, the SNV at this site was not detected by the shotgun approach due to the low frequency of the alternative allele, despite the sufficient SNV probability assigned by the algorithm.

The most significant differences between the two approaches were observed at site 7065, where SNV was not detected in 15 accessions in the targeted approach. On manual inspection of the mapped reads in the targeted approach, we observed that the consensus sequences of all 15 accessions had a polymorphism that was not determined due to an underestimation of SNV probability. We assume that the lower probability of polymorphism assigned by the tool was due to the locus being located immediately adjacent to a homopolymeric adenine region, which resulted in reduced quality of nucleotide determination at this site. One of the biggest challenges of Ion Torrent sequencing technology is the accuracy of recognizing the number of identical nucleotides (homopolymers). As the length of the homopolymer increases, it becomes more difficult to deduce the number of bases from the signals, and this can lead to an error in determining the sequence [22].

Contrary to the previous examples, at site 33406, the SNV was detected in three accessions (ALB-38AI, HRV-749 and SLO-121S) using the targeted approach, and it was not detected by the shotgun approach. The locus is near the homopolymeric region of thymines, as in the case of site 7065, which may affect the quality of the nucleotide identified at this site due to the shortcomings of Ion Torrent technology [60,61]. As reported in other studies [44], a polymorphism at position 33406 was detected in the cultivar ‘Zibibbo’; the same cultivar is also represented in our set of accessions by the accession HRV-749. Cv. Zibibbo can also be found under the name ‘Muscat of Alexandria’, which is represented in our set of accessions by the accession SLO-121S. For the accession ALB-38AI (Rrumbullak i vonë) we did not find the synonymous name in the literature. The discovery of such rare SNVs in synonymous varieties thus confirms the appropriateness of the approach. Based on obtained results, we manually reviewed the mapped reads of shotgun approach at site 33406 in accessions ALB-38AI, HRV-749 and SLO-121S. We found that an alternative nucleotide was identified in the consensus sequence, but the SNV as such was not detected by our analysis procedure due to the stringent variant detection conditions. Specifically, the frequency of the alternative allele was not exceeded at this locus in any of the three accessions. The frequency of the alternative allele at the site was 44% in the ALB-38AI accession, 56% in the SLO-121S accession and 40% in the HRV-749 accession. In the latter case, the coverage was also below the threshold of 20 reads, which is lower than the minimum cutoff of the 25 reads that we had established. For all three accessions, the tool estimated the probability of a variant at this site to be 1.00, suggesting that we probably set the threshold too high in the first round of variant detection. Based on the literature, we assume that the frequency was also reduced due to the locus being located immediately adjacent to a homopolymeric region, which can lead to errors in nucleotide determination at that site [60,61].

To determine haplotypes from alternative reference sequences, we used three known polymorphic sites that have already been shown to be informative in several grapevine studies [36,45,62]. All three selected sites are located in non-coding regions. Site 205 in the *V. vinifera* cpDNA is located between the *trnH* and *psbA* genes, while sites 86715 and 86721 are positioned within the intron of the *rpl16* gene. These two intronic sites have previously been used for taxonomic species differentiation at the molecular level [63,64,65]. Most studies on chloroplast DNA (cpDNA) haplotypes in grapevine have relied on microsatellite markers rather than SNV markers. In their landmark study, Arroyo-García et al. [66] identified a total of eight different haplotypes based on chloroplast microsatellite markers (A–H). Among these, haplotypes A, B, C, and D were shown to be the most common. Haplotype A is mainly characteristic of the Iberian Peninsula, B of Eastern Europe, C of Central Europe and North Africa, and D of the Apennine and Balkan Peninsulas. In our study, we identified three haplotypes among the analyzed accessions, namely ATT, ATA, and GTA, based on specific single nucleotide variants rather than microsatellite markers (Figure 4). Marker systems based on cpSNVs and cpSSRs differ fundamentally in their mutation mechanisms and resolution and therefore should not be directly equated when comparing haplotypes. Of the three haplotypes identified in our study, GTA is the least frequent, occurring in 16.6% of all accessions. It was not detected in the Macedonian and Montenegrin varieties, which may reflect either the true absence of this haplotype in these populations or the limited accession size from these two groups. The GTA haplotype was most highly represented with the accessions from French collection, with 2 from Georgia (FRA-7F, FRA-28F), 2 from Portugal (FRA-19F, FRA-20F), and 4 from France (FRA-13F, FRA-16F, FRA-22F, FRA-33F). The fourth haplotype, AAA, has been reported in *sativa* and *sylvestris* subspecies originating from Georgia and Transcaucasia, respectively [36,67]. In the studies [45,62], where the diversity of chloroplast DNA in different cultivars of *V. vinifera* L. was investigated, it was also found that ATT, ATA, and GTA haplotypes are present in geographically distinct regions.

Phylogenetic relationships based on the chloroplast genome are of unique value for understanding the origin of plants [68]. But due to its uniparental inheritance of cpDNA and limited resolution, chloroplast DNA provides only a partial view of grapevine evolutionary history and should be interpreted as complementary to nuclear genomic data. In the present study, the phylogenetic tree clearly shows three clusters corresponding to three specific haplotypes. As expected, due to the high similarity of the analyzed sequences, we have many branches in the tree with a bootstrap value (BV) below 70. Bootstrap values above 70 reflect at least a 95% probability that the corresponding phylogenetic group is correctly supported [69]. In our tree, BVs above 70 were observed at branch points separating clusters of three haplotypes. Only the accessions SLO-45S and SLO-92S deviated from this pattern, corresponding to a relatively low clustering error of approximately 2%. The phylogenetic network also revealed three clearly separated clusters of nodes, demonstrating that it successfully distinguished alternative sequences corresponding to the three selected haplotypes (Figure 6). Despite widespread use of phylogenetic trees, due to their simple structure, they have some disadvantages, such as an inability to represent more complex evolutionary mechanisms correctly like hybridization and genome rearrangements. Phylogenetic networks are an alternative that can better demonstrate such phenomena [70].

## 4. Materials and Methods

### 4.1. Plant Materials and DNA Extraction

Plant material consisted of 409 grapevine accessions obtained from national and institutional grapevine collections maintained by the project partners. All analyzed accessions represent cultivated grapevine material conserved in national and international germplasm collections. As the accessions originate from multiple collections, collection-specific accession codes were used as identifiers; full details of cultivar names, countries of origin, accession codes and the list of contributors responsible for the plant material from each country are given in Supplementary Table S1 accession. Six accessions (Barbera, Cabernet, Chardonnay, Merlot, Pinot, and Sultanine) were included in this study as reference varieties. The remaining accessions originated from different countries, with 38 from Albania, 56 from Bosnia and Herzegovina, 85 from Croatia, 26 from Greece, 6 from Macedonia, 17 from Montenegro, 122 from Slovenia, and 28 from Serbia. Twenty-five accessions assigned as FRA are not exclusively French accessions, but represent a highly heterogeneous set of accessions held by the French National Institute for Agronomic Research (INRAE, Institut national de la recherche agronomique) originating from France (7), Hungary (3), Italy (3), Georgia (2), Armenia (2), Portugal (2), Romania (1), Greece (1), Russia (1), Iran (1), Afghanistan (1), and Uzbekistan (1).

DNA was isolated from young leaves of sprouting grapevine plants. Genomic DNA was extracted using a cetyltrimethyl ammonium bromide (CTAB) method [71]. The concentration of DNA was checked using a Nanovue Plus (GE HealthCare, Chicago (IL), USA.

### 4.2. Preparation of Sequence Libraries

Sequence library preparation followed protocol [72], with modifications introduced in our laboratory. In brief, the first step was to cut the DNA by means of sonication. The cutting performance was qualitatively checked by electrophoresis on a 1% agarose gel. DNA digestion was considered successful if the bulk of the DNA was in the 100–300 bp size range. After cutting, the sticky ends of the cut DNA were repaired by T4 DNA polymerase (NEB, Ipswich, MA, USA, cat. no. M0203), and barcoded adapters were ligated and nick repaired to each DNA accession using T4 DNA ligase (NEB, cat. no. M0202) and Bst 2.0 WarmStart^®^ DNA polymerase (NEB, cat. No.M0538). All ligated products were pooled and cleaned with magnetic beads (Magtivio, Daelderweg, The Netherlands, cat no. MDKT00010075). The adapter P1 was added to all accessions, and the adapters A were barcoded differently for each accession to allow multiplexing. Two oligonucleotide primers (P1amp: 5′CCACTACGCCTCCGCTTTCCTCTCTATG3′ and T_PCR_A: 5′CCATCTCATCCCTGCGTGTC3′), complementary to both adaptors, were used for library amplification in PCR. The prepared and purified libraries were quantified by on-chip electrophoresis using an Agilent Bioanalyzer 2100 (Agilent Technologies, Santa Carla, CA, USA).

### 4.3. Target Capture of cpSNV Sites

Seven cpDNA probes (Supplementary Table S8), each containing one known SNV, were enriched using streptavidin-labeled magnetic beads and biotin-labeled DNA probes designed by Arbor Biosciences (Ann Arbor, MI, USA). The probes were either 100 nt or 120 nt in length and were designed so that a known polymorphic site overlapped with the middle of the probe. The capturing procedure followed the manufacturer’s instructions [73].

The sequencing of whole genomes and captured libraries was performed on an Ion PI v3 chip in an Ion Proton Sequencer (Life Technologies, Carlsbad, CA, USA).

### 4.4. Bioinformatic Analysis of Sequencing Results

The bioinformatics analysis of the sequence data was based on the CLC Genomics Workbench (CLC GW 20.0.4) [74], software that integrates a number of NGS bioinformatics tools, e.g., read mapping tools, genetic variant detection tools and a set of phylogenetic tools. The remaining steps of the analysis were performed with Python 3.8 (Python Software Foundation, Wilmington, DE, USA) and R scripts [75]. The ideogram images were created with the tool Circos (v069.9) [76].

#### 4.4.1. Mapping Reads to the Chloroplast Genome

The mapping of the reads was performed with the map reads as a reference tool in CLC GW. Sequencing reads were mapped to the reference chloroplast genome *Vitis vinifera* ‘Maxxa’ (NCBI RefSeq accession number NC_007957.1) and 12× assembly of Pinot Noir nuclear genome (GCA_000003745.2).

#### 4.4.2. Variant Calling and Filtering

Variant identification from the mapping profiles was performed in two sequential rounds of detection. In the first round, mapped reads (BAM files) were analyzed using the Fixed Ploidy Variant Detection tool (v2.0.3) in CLC Genomics Workbench with haploid ploidy, exclusion of nonspecific reads, removal of variants located in homopolymeric regions ≥3 bp, and a minimum variant probability of 90%. Structural variants and indels/deletions were not considered because such variants in chloroplast genomes occur predominantly in polynucleotide (homopolymer) tracts, which are also associated with inaccurate base calling in Ion Torrent sequencing data [77,78]. The resulting variants were further filtered to retain only SNVs with a coverage ≥ 25 reads and a frequency ≥ 80%, while variants within the inverted repeat regions of the chloroplast genome were removed. This step produced a conservative set of reliable variant positions. In the second round, the same detection tool was applied with relaxed settings, using only the minimum probability threshold of 90%, generating a broader list of candidate variants. The final set of chloroplasts SNVs was then obtained by retaining only those variants occurring at the high-confidence positions identified in the first round, resulting in a curated and robust dataset for downstream analyses.

The variants found by the shotgun sequencing approach in each of the 409 accessions were used to generate so-called alternative reference sequences of chloroplast DNA. This step was performed using the FastaAlternateReferenceMaker (v4.1.8.1) tool found in the GATK genomic analysis toolkit [79].

#### 4.4.3. Haplotype Analysis

Using the variant data detected by the shotgun approach, we assigned a haplotype to each accession according to the three loci in the chloroplast genome. These are positions 205, 86715, and 86721, which have been shown in several studies to be informative polymorphic sites in cpDNA [33,37,38]. The representation of haplotypes according to country of origin was analyzed by Fisher’s exact test with a 95% confidence level. The test was performed using a script written in the R programming language (v4.0.1) to test the following null hypothesis: The representation of a haplotype is independent of the country of origin.

#### 4.4.4. Phylogenetic Analysis

Alternative reference sequences were aligned using MAFFT (v7.471) [80] and a phylogenetic tree was constructed using RAxML (v8.0.0) [81] using a maximum likelihood (ML) approach. The tree was displayed using the R library ggtree (v2.4.1) [82]. To construct the phylogenetic network, the aligned sequences were converted to NEXUS format using the Python library Biopython (v1.78) [83]. In this format, the sequences were imported into the PopART program (v1.7), which was used to construct the network using the merge-means method. This was then displayed graphically using Gephi (v0.9.2) [84].

## 5. Conclusions

Comparison of the seven SNV loci between the targeted and shotgun approaches showed very high concordance. Out of all accession–locus comparisons across 409 accessions, only 20 instances of discordance were detected, and these were limited to five loci/sites (7065, 33406, 123664, 123690, and 128420). Most discrepancies occurred at loci affected by low capture performance or known homopolymer-associated calling issues, indicating that the overall agreement between the methods is robust. As the validation process has identified the causes of discrepancies between the two approaches, further optimization of the parameters and setting the thresholds for SNV detection in the shotgun approach can improve the process. This study represents the most extensive investigation of grapevine chloroplast genomes conducted to date. We sequenced the chloroplasts of 409 cultivated *Vitis vinifera* accessions and systematically identified single nucleotide variant (SNV) polymorphisms, providing an unprecedented resource for studies of genetic diversity and grapevine phylogeny. Chloroplast SNV data are expected to be particularly valuable for future parentage and pedigree analyses, where maternal lineage information represents a critical component. Future research will therefore focus on integrating chloroplast and nuclear SNP datasets and applying combined organellar and nuclear approaches to more comprehensively investigate lineage relationships, parentage, and clonal diversification in grapevine.

## Figures and Tables

**Table 1:** Chloroplast SNVs detected by targeted sequencing and their frequencies in cultivated *V. vinifera* accession.

Locus	SNP Position	Reference Allele	Alternative Allele	Proportion of Samples with Alternative Allele [%]
SNP_NG_C_003	7065	C	T	32.52
SNP_NG_AS_001	33406	A	C	0.73
SNP_NG_D_003	73765	G	A	47.19
SNP_NG_C_001	75398	C	T	33.25
SNP_NG_C_002	123664	T	G	36.67
SNP_NG_NVD_001	123690	C	A	46.94
SNP_NG_D_001	128420	C	T	46.94

## Data Availability

The datasets generated and analyzed during the current study are available in the NCBI Sequence Read Archive (SRA) repository (https://www.ncbi.nlm.nih.gov/sra/, accessed on 1 December 2025) under the BioProject accession number PRJNA1366753. The data are not officially released yet; the reviewer link for BioProject and associated SRA metadata are available at https://dataview.ncbi.nlm.nih.gov/object/PRJNA1366753?reviewer=pql2i9lp7e4kshurjbrel2nouv, accessed on 21 December 2025) in read-only format.

## Supplementary Materials

The following supporting information can be downloaded at https://www.mdpi.com/article/10.3390/ijms27031583/s1.
