# Supplementary material for: High-Resolution Chloroplast SNV Profiling of 409 Grapevine (Vitis vinifera L.) Cultivars Using Whole-Genome Shotgun Sequencing
Source: Int J Mol Sci. 2026 Feb 5;27(3):1583. doi: 10.3390/ijms27031583 (PMC12898280; doi:10.3390/ijms27031583)
Supplement: Supplementary file 1 [file ijms-27-01583-s001.zip › ijms-4087053-supplementary/Tables S1, S3-S9.pdf]

Table S1. List of cultivated *Vitis vinifera* L. accessions analysed in this study. All accessions represent cultivated grapevine genotypes; wild grapevine (*Vitis vinifera* subsp. *sylvestris*) was not included. Country codes are as follows: (ALB, Albania; BIH, Bosnia and Herzegovina; FRA, France; GRE, Greece; HRV, Croatia; MKD, North Macedonia; MNE, Montenegro; SLO, Slovenia; SRB, Serbia; IRN, Iran; HUN, Hungary; GEO, Georgia; ROU, Romania; ARM, Armenia; ITA, Italy; PRT, Portugal; RUS, Russia; UZB, Uzbekistan; AFG, Afghanistan). The six samples representing the reference varieties are written in *italics* (REF-1 to REF-6). Sample providers (co-authors) are: Denis Rusjan (SLO); Tatjana Jovanović-Cvetković and Ana Mandić (BIH); Aida Dervishi (ALB); Dragoslav Ivanišević (SRB); Katerina Binari (GRE); Klime Beleski (MKD); Vesna Maraš (MNE); Goran Zdunić (HRV); Roberto Bacilieri (FRA).

Accession (Cultivar Name)	Country of Origin	Collection-Specific Accession Code	Accession (Cultivar Name)	Country of Origin	Collection-Specific Accession Code
Kallmet	ALB	ALB-1A	Debejan	HRV	HRV-125
Debinë e bardhë	ALB	ALB-2A	Magrovina	HRV	HRV-126
Vlosh	ALB	ALB-3A	Žumić	HRV	HRV-127
Shesh i bardhë	ALB	ALB-4A	Belina istarska	HRV	HRV-128
Shesh i zi	ALB	ALB-5A	Borgonia	HRV	HRV-129
Pulez	ALB	ALB-6A	Plavinica	HRV	HRV-131
Tajgë e zezë	ALB	ALB-7A	Crna pergola	HRV	HRV-132
Serine e bardhë	ALB	ALB-8A	Borgonja bijela	HRV	HRV-133
Debinë e zezë	ALB	ALB-9A	Plavčina	HRV	HRV-118
Kryqez	ALB	ALB-10A	Malvasia lunga	HRV	HRV-710
Viktoria	ALB	ALB-11A	Gustopupica ninska	HRV	HRV-711
Perla	ALB	ALB-12A	Mladenka	HRV	HRV-715
Muskat	ALB	ALB-13A	Moscato rosa	HRV	HRV-716
Merlot	ALB	ALB-15A	Malvasia dubrovačka	HRV	HRV-708
Tribian	ALB	ALB-16A	Dobričić	HRV	HRV-722
Shesh i zi	ALB	ALB-17A	Plavac mali	HRV	HRV-727
Zvernac	ALB	ALB-19A	Maraština Omiš	HRV	HRV-740
Hibrid	ALB	ALB-20A	Pošip crni	HRV	HRV-706
Qelibar	ALB	ALB-2AI	Cetinka	HRV	HRV-719
Potek e zezë	ALB	ALB-9AI	Babica Crna	HRV	HRV-720
Tajgë lici	ALB	ALB-10AI	Chardonnay	HRV	HRV-724
Durrsak i bardhë	ALB	ALB-11AI	Frmentum	HRV	HRV-728
Debinë leksoviku	ALB	ALB-17AI	Prč Bijeli	HRV	HRV-743
Dimërak	ALB	ALB-18AI	Plava lovora	HRV	HRV-707
Debinë përmeti	ALB	ALB-19AI	Rogoznička	HRV	HRV-718
Tajgë e bardhë	ALB	ALB-20AI	Tsitsa kaprei (Čilibarka)	MKD	MKD-401
Stambolleshë	ALB	ALB-21AI	Manastirko belo	MKD	MKD-402
Kosinjot	ALB	ALB-32AI	Ohridsko crno	MKD	MKD-403
Jediveren	ALB	ALB-33AI	Koncanka	MKD	MKD-404
Gomaresh	ALB	ALB-34AI	Kadarka	MKD	MKD-405
Sinabel	ALB	ALB-35AI	Crn valandovski drenak	MKD	MKD-406
Meresnik	ALB	ALB-37AI	Razaklija (Gornji Brčeli)	MNE	MNE-201
Rrumbullak i vonë	ALB	ALB-38AI	Vranac (Gornji Brčeli)	MNE	MNE-202
Kazarka	ALB	ALB-39AI	Kratošija (Gornji Brčeli)	MNE	MNE-203
Laracik	ALB	ALB-41AI	Lisica 1 (Gornji Brčeli)	MNE	MNE-204
Caushverdhë	ALB	ALB-42AI	Lisica 2 (Gornji Brčeli)	MNE	MNE-206
I bardhi cipëhollë	ALB	ALB-43AI	Lisica (Sotonići)	MNE	MNE-207
I bardhi cipëfortë	ALB	ALB-44AI	Zadrimka (Boljevići)	MNE	MNE-208
Trnjak	BIH	BIH-511	Lisica (Boljevići)	MNE	MNE-209
Plavica	BIH	BIH-514	Kratošija 1 (Komani)	MNE	MNE-210
Iknica	BIH	BIH-515	Vranac 1 (Komani)	MNE	MNE-211
Bjeli prošip	BIH	BIH-516	Kratošija 2 (Komani)	MNE	MNE-212

Žestac	BIH	BIH-517	Kadarun (Gornji Morinj)	MNE	MNE-213
Podbjel	BIH	BIH-518	Čubrica (Kosor)	MNE	MNE-214
Šljiva	BIH	BIH-519	Prokupac (Ubli)	MNE	MNE-215
Bena	BIH	BIH-520	Krstač 1 (Piperi)	MNE	MNE-216
Lipanjka	BIH	BIH-521	Vranac 2 (Komani)	MNE	MNE-217
Šljiva crna	BIH	BIH-522	Krstač 2 (Piperi)	MNE	MNE-218
Crni prošip	BIH	BIH-523	Modra frankinja	SLO	SLO-1
Ruža	BIH	BIH-524	Kraljevina	SLO	SLO-2
Žlozder	BIH	BIH-525	Kerner	SLO	SLO-3
Medenka	BIH	BIH-526	Žametna črnina	SLO	SLO-6
Radovača vučja	BIH	BIH-601	Biserka	SLO	SLO-2S
Zelena žilavka	BIH	BIH-602	Šalamenci	SLO	SLO-4S
Alikant buše	BIH	BIH-609	Barbera BAVCON	SLO	SLO-8S
Zelena žilavka	BIH	BIH-302	Ranfol AV	SLO	SLO-9S
Žilavka Ortiješ	BIH	BIH-303	Medena Glera	SLO	SLO-11S
Stara blatina	BIH	BIH-305	Sultanina	SLO	SLO-13S
Kadarun vučja	BIH	BIH-307	Rebula AV	SLO	SLO-14S
Drenak vučja	BIH	BIH-308	Plovdina	SLO	SLO-18S
Surac Đ. Vujović	BIH	BIH-309	Modra frankinja	SLO	SLO-19S
Radovača vučja	BIH	BIH-311	Cipro AV	SLO	SLO-21S
Blatina bruna Mostar	BIH	BIH-313	Zeleni silvanec	SLO	SLO-23S
Kadarun Đ. Vujović	BIH	BIH-316	Šipon	SLO	SLO-25S
Radovača	BIH	BIH-501	Črešnjiška črnina	SLO	SLO-27S
Dugoljak	BIH	BIH-502	Planinka	SLO	SLO-28S
Ćevruša	BIH	BIH-503	Vrtovka	SLO	SLO-29S
Ružica	BIH	BIH-504	Daniela	SLO	SLO-30S
Uzbrdnača	BIH	BIH-506	Istarska malvazija	SLO	SLO-31S
Elezuša	BIH	BIH-507	Trevolina	SLO	SLO-32S
Oručevka	BIH	BIH-508	Rdeča žlahtnina	SLO	SLO-33S
Crno	BIH	BIH-509	Rumeni plavec	SLO	SLO-34S
Nadiđar	BIH	BIH-510	Bela žlahtnina	SLO	SLO-35S
Krkošija	BIH	BIH-512	Zdenka	SLO	SLO-37S
Surac	BIH	BIH-513	Bontempa	SLO	SLO-39S
Mala blatina	BIH	BIH-304	Egiptarka	SLO	SLO-40S
Plavka Mostar	BIH	BIH-306	Zelen	SLO	SLO-41S
Rezaklija Lastva	BIH	BIH-310	Pinela	SLO	SLO-42S
Plavka buna Mostar	BIH	BIH-314	Barbera	SLO	SLO-43S
Alikante buše	BIH	BIH-315	Ranfol	SLO	SLO-44S
Radovača Lastva 1	BIH	BIH-318	Ranina	SLO	SLO-45S
Menigovka	BIH	BIH-320	Verdasa	SLO	SLO-46S
Radovača Lastva 2	BIH	BIH-312	Kanarjola	SLO	SLO-47S
Žilavka M.M.	BIH	BIH-301	Duraniia	SLO	SLO-49S
Dobrogostinja	BIH	BIH-319	Izolana	SLO	SLO-51S
Mala blatina	BIH	BIH-608	Belina Pregara	SLO	SLO-54S
Surac bijeli Đ. Vujović	BIH	BIH-317	Istarska belina	SLO	SLO-55S
Menigovka	BIH	BIH-603	Belina drobna	SLO	SLO-56S
Surac Đ. Vujović	BIH	BIH-604	Bianchera	SLO	SLO-57S
Trnjak	BIH	BIH-605	Rečigla	SLO	SLO-58S
Radovača	BIH	BIH-606	Peteršiljasta žlahtnina	SLO	SLO-59S
Rezaklija	BIH	BIH-607	Sevka	SLO	SLO-64S
Surac	BIH	BIH-610	Rebula stara	SLO	SLO-66S
Žlozder	BIH	BIH-611	Pivka Brda	SLO	SLO-68S
Siah	IRN	FRA-3F*	Zelenika AV	SLO	SLO-70S

Furmint	HUN	FRA-6F*	Verduc	SLO	SLO-72S
Avassirkhva	GEO	FRA-7F*	Zunek	SLO	SLO-73S
Razachie rosie	ROU	FRA-9F*	Cencukna (Brda)	SLO	SLO-75S
Ganziandy	ARM	FRA-10F*	Pikolit (IT)	SLO	SLO-77S
Nuragus	ITA	FRA-11F*	Cohovka	SLO	SLO-78S
Riesling	FRA	FRA-13F*	Drenik	SLO	SLO-79S
Mondeuse	FRA	FRA-16F*	Pergolin 1	SLO	SLO-80S
Olivette noire	FRA	FRA-18F*	Sušc	SLO	SLO-81S
Alfrocheiro Tinto	PRT	FRA-19F*	Rogozdnica	SLO	SLO-82S
Espadeiro tinto	PRT	FRA-20F*	Cividin	SLO	SLO-83S
Mancin	FRA	FRA-22F*	Klarnica	SLO	SLO-84S
Olivette noire	FRA	FRA-23F*	Belina debela	SLO	SLO-85S
Perle de Csaba	HUN	FRA-24F*	Tržaška	SLO	SLO-86S
Badagui noir	GEO	FRA-28F*	Medena glera Panelca	SLO	SLO-87S
Foglia Tonda	ITA	FRA-30F*	Teran Fakin 1 Poreč	SLO	SLO-1T
Grillo	ITA	FRA-31F*	Teran Fakin 2 Poreč	SLO	SLO-2T
Perle de Csaba	HUN	FRA-32F*	Legović IPTIPO	SLO	SLO-3T
Mondeuse	FRA	FRA-33F*	Terrano ISV F2	SLO	SLO-4T
Tzimliansky belyi	RUS	FRA-34F*	Teran 32 Poreč	SLO	SLO-5T
Vassagra bielaia	UZB	FRA-8AF*	Maločrn	SLO	SLO-6T
Cabernet franc	FRA	FRA-11AF*	Pagadebit	SLO	SLO-7T
Ganziandy	ARM	FRA-14AF*	Bela istrska	SLO	SLO-9T
Sultanine monococco	GRE	FRA-18AF*	Refosco Faedis	SLO	SLO-10T
Golodan	AFG	FRA-19AF*	Refosco Ped Roso	SLO	SLO-12T
Mandilaria	GRE	GRE-1AG	Refoscone	SLO	SLO-13T
Dafni	GRE	GRE-2AG	Refosco di Rauscedo	SLO	SLO-14T
Liatiko	GRE	GRE-3AG	Refošk grbec Pouzelca	SLO	SLO-15T
Mavrotragano 18 20	GRE	GRE-4AG	Refošk abitanti Pouzelca	SLO	SLO-16T
Fokiano	GRE	GRE-5AG	Piranski refošk Pouzelca	SLO	SLO-17T
Limniona	GRE	GRE-6AG	Refošk Pouzelca	SLO	SLO-18T
Limnio	GRE	GRE-7AG	Teran Istra Pouzelca	SLO	SLO-19T
Mavrotragano 36	GRE	GRE-8AG	Refošk Šepulje	SLO	SLO-20T
Agianiotiko	GRE	GRE-9AG	Sladki teran	SLO	SLO-21T
Ritino	GRE	GRE-10AG	Pinjola	SLO	SLO-22T
Rombola	GRE	GRE-801	Refošk SI 35	SLO	SLO-23T
Pavlos	GRE	GRE-802	Istrski refošk	SLO	SLO-11T
Plito	GRE	GRE-805	Pokov zelen	SLO	SLO-89S
Platani	GRE	GRE-809	Števerjana	SLO	SLO-90S
Areti	GRE	GRE-812	Sladki teran	SLO	SLO-91S
Kontokladi	GRE	GRE-813	Virbina	SLO	SLO-92S
Makripodia	GRE	GRE-810	Dišečka	SLO	SLO-93S
Mosxoudi	GRE	GRE-804	Rožca	SLO	SLO-94S
Athiri	GRE	GRE-815	Pokalca Golo Brdo	SLO	SLO-97S
Katsano	GRE	GRE-807	Dolga petlja	SLO	SLO-100S
Triferopodia	GRE	GRE-811	Cundra	SLO	SLO-101S
Thrapsathiri	GRE	GRE-806	Volovnik	SLO	SLO-102S
Thrapsathiri 2	GRE	GRE-816	Guštana	SLO	SLO-103S
Dafnia	GRE	GRE-814	Pergolin 2	SLO	SLO-104S
Arkadino	GRE	GRE-808	Vitovska grganja	SLO	SLO-105S
Vilana	GRE	GRE-803	Racuk	SLO	SLO-108S
Teran bijeli	HRV	HRV-130	Pika AV	SLO	SLO-110S
Cipar	HRV	HRV-701	Glera I	SLO	SLO-113S
Palaruša	HRV	HRV-702	Sladkočrn	SLO	SLO-116S

Palagružanka	HRV	HRV-703	Aleksandrijski muškat	SLO	SLO-121S
Okatac Omiš	HRV	HRV-704	Muškat petit grain	SLO	SLO-123S
Svrdlovina	HRV	HRV-705	Žametovka Lent	SLO	SLO-125S
Muškat ruža omiški	HRV	HRV-709	Lipovina	SLO	SLO-69S
Bogdanuša	HRV	HRV-712	Plavina Pouzelca	SLO	SLO-127S
Vrškajica	HRV	HRV-713	Črešnjiška črnina	SLO	SLO-128S
Silibijanac	HRV	HRV-714	Gnjet	SLO	SLO-129S
Krkošija	HRV	HRV-717	Maločrn 4	SLO	SLO-130S
Zlatarica	HRV	HRV-721	Črna borgonja	SLO	SLO-132S
Plavac sivi	HRV	HRV-723	Cesarski pen	SLO	SLO-137S
Rudežuša	HRV	HRV-725	Šipelj	SLO	SLO-138S
Bombino bianco	HRV	HRV-726	Refošk R3	SLO	SLO-R3
Crljenak kaštelanski 091	HRV	HRV-729	Refošk križanec R17	SLO	SLO-R17
Šarica trišnjavica	HRV	HRV-730	Refošk R19	SLO	SLO-R19
Marinkovića grozje	HRV	HRV-731	Refošk Brda 1	SLO	SLO-186S
Debit	HRV	HRV-732	Beli teran 1	SLO	SLO-187S
Ninčuša	HRV	HRV-733	Teran krom. 1	SLO	SLO-188S
Karstičevica	HRV	HRV-734	Teran Istra 2	SLO	SLO-189S
Fiano	HRV	HRV-735	S. teran 1	SLO	SLO-190S
Bratkovina Čara	HRV	HRV-737	Laški rizling	SLO	SLO-4
Gegić	HRV	HRV-738	Rebula 100 let	SLO	SLO-67S
Lasina	HRV	HRV-739	Poljšakica	SLO	SLO-74S
Zlatarica vrgorska	HRV	HRV-741	Ružica crvena	SRB	SRB-16
Žilavka	HRV	HRV-742	Drenjak crni	SRB	SRB-17
Plavina	HRV	HRV-744	Kadarka bela	SRB	SRB-24
Xinomavro	HRV	HRV-745	Žunić	SRB	SRB-8
Vugava Vis	HRV	HRV-746	Muskat krokan	SRB	SRB-9
Tanetova loza	HRV	HRV-747	Drenjak crveni	SRB	SRB-10
Zibibo	HRV	HRV-749	Urban crveni	SRB	SRB-11
Tempranillo	HRV	HRV-751	Bele kozijse sise	SRB	SRB-12
Bak	HRV	HRV-753	Kokur beli	SRB	SRB-13
Beretinjok bijeli	HRV	HRV-754	Kadarun	SRB	SRB-14
Pošip sitni bijeli	HRV	HRV-755	Bagrina	SRB	SRB-15
Grk	HRV	HRV-756	Tamjanika crna	SRB	SRB-18
Okatica bijela	HRV	HRV-760	Kavčina	SRB	SRB-19
Cima rosa	HRV	HRV-101	Muskat ruža	SRB	SRB-20
Muškat momjanski	HRV	HRV-102	Beli medenac	SRB	SRB-21
Vela pergola	HRV	HRV-103	Trbljan beli	SRB	SRB-22
Hrvatica	HRV	HRV-104	Kadarka	SRB	SRB-23
Trojščina	HRV	HRV-105	Ružica mirisava	SRB	SRB-25
Duranija	HRV	HRV-106	Smederevka	SRB	SRB-26
Dolcin	HRV	HRV-107	Suralisičina	SRB	SRB-27
Brajdica	HRV	HRV-108	Čauš crveni	SRB	SRB-28
Surina	HRV	HRV-109	Drenak beli	SRB	SRB-29
Teran	HRV	HRV-110	Ćilibarka	SRB	SRB-30
Pošip	HRV	HRV-111	Krivalj	SRB	SRB-31
Pagadebit istarski	HRV	HRV-112	Prokupac	SRB	SRB-32
Muškat ruža	HRV	HRV-113	Plodovina crna	SRB	SRB-33
Garganja	HRV	HRV-114	Sremska zelen	SRB	SRB-34
Malvazija istarska	HRV	HRV-115	Bela dinka	SRB	SRB-7
Jarbola	HRV	HRV-116	*Barbera*		REF-1
Bilan bijeli	HRV	HRV-117	*Cabernet*		REF-2
Plavica	HRV	HRV-119	*Chardonnay*		REF-3

Sušac	HRV	HRV-121	*Merlot*	REF-4
Volarovo	HRV	HRV-122	*Pinot*	REF-5
Ošljevina	HRV	HRV-123	*Sultanine*	REF-6
Rušljan	HRV	HRV-124		

* Accessions originating from INRAE French collection. Reference varieties (REF-1 to REF-6) are included for benchmarking purposes and are not assigned country codes.

Table S3. List of 93 SNVs detected in chloroplast genomes of 409 cultivated *V. vinifera* accessions.

SNP Position	Reference Allele	Alternative Allel	Share of Samples [%]	Amino Acid Change	Gene
4547	A	T	47,19		
5591	A	C	47,19		rps16
5978	T	G	35,94		rps16
7065	C	T	36,19		
8114	A	G	83,37		
8175	T	C	83,37		
9996	A	G	82,89		
10017	T	A	40,59		
10720	G	A	1,71		trnG-GCC
10727	G	T	0,24		trnG-GCC
11120	A	G	80,93		trnG-GCC
11625	T	A	76,53		
14416	A	G	83,37		atpF
14794	T	A	76,77		
17422	G	T	1,71	Thr175Lys	rps2
20840	C	T	83,37	Ser503Asn	rpoC2
21046	G	T	83,37	Phe434Leu	rpoC2
22321	A	G	83,37		rpoC2
24437	C	A	83,37		rpoC1
28707	T	G	35,70		
29232	T	C	0,49		
29444	T	G	0,73		
29507	C	T	83,37		
29571	G	T	83,37		
30783	T	C	83,37		
30929	T	G	1,71		
32670	C	G	83,37		
34037	C	A	83,37		
36031	C	A	47,19		
36397	A	C	83,37		psbD
39357	T	A	83,37		psbZ
39679	A	T	92,18		
39681	A	T	95,11		
39883	T	A	60,39		
39885	C	T	74,57		
39961	T	G	82,64		
42474	C	T	78,48	Gly245Ser	psaB
43685	T	G	46,94		psaA
43910	C	G	47,19		psaA
47786	A	G	1,71		ycf3
50339	T	G	46,94		
50627	T	A	81,42		
51700	A	T	33,25		trnL-UAA

54900	T	G	0,24		
54999	T	G	35,94		
55094	T	C	83,37		
59143	A	T	47,19		
61186	T	G	83,37		
63186	C	T	83,37		
63478	T	A	81,66		
63819	A	G	83,37		dog
67651	T	C	83,37		
70248	A	C	36,19		
70595	C	T	83,37		
70921	G	A	36,19		
71588	C	T	82,40		
73579	C	T	0,49		rpl20
73765	G	A	47,19		rpl20
75398	C	T	33,25		clpP
77099	C	T	83,37		
80022	A	C	83,37		petB
80194	C	T	46,94		petB
80276	C	T	83,37		petB
82561	T	G	0,98		
85961	G	T	0,24		
86721	A	T	46,45		rpl16
87287	A	C	0,24		rpl16
89112	C	T	46,94		rps19
117310	T	G	83,37		ndhF
119471	A	C	0,24		
119481	G	T	83,37		
119571	T	G	83,37		
121659	C	G	1,71		
121732	A	C	83,13		
122363	T	A	0,49	Leu301Phe	ndhD
123321	A	T	77,51		
123664	T	G	36,19		
123690	C	A	47,19		
124258	G	A	83,37		
125681	G	A	83,37		ndhI
126020	G	T	47,19	Leu331Met	ndhA
128420	C	T	47,19	Glu302Lys	ndhH
130537	T	G	1,71	Ile1716Leu	ycf1
131820	T	C	83,37	Gln1288Arg	ycf1
133211	T	G	35,94		ycf1
133359	A	C	48,66	Leu775Trp	ycf1
133934	G	T	83,37		ycf1
133982	A	C	33,25		
205	G	A	83,37		ycf1
1552	G	A	0,49	Ile567Met	
4190	C	T	1,71		
4444	T	G	1,71		trnK-UUU
4527	T	G	33,25		

Table S4. Contingency table of the number of samples by haplotype and country of origin.

Country/Haplotype	ALB	BIH	CRO	FRA	GRE	MKD	MNE	SLO	SRB	Total
**ATA**	13	29	35	10	2	4	5	33	17	148
**ATT**	24	23	32	7	20	2	12	60	8	188
**GTA**	1	5	18	8	4	0	0	28	3	67
**Total**	38	57	85	25	26	6	17	121	28	403

Table S5. Calculated *p-values* for the 108 Fisher tests performed.

	ALB:BIH	ALB:HRV	ALB:FRA	ALB:GRE	ALB:MKD	ALB:MNE
ATA:ATT	0,252729	0,281856	0,329499	0,14634	0,384771	0,9816
ATA:GTA	0,930214	0,193238	0,170847	0,107475	1	1
ATT:GTA	0,384771	0,036058	0,016379	0,384771	1	1
	ALB:SLO	ALB:SRB	BIH:HRV	BIH:FRA	BIH:GRE	BIH:MKD
ATA:ATT	1	0,108316	0,95362	1	**0,01129**	0,943243
ATA:GTA	0,066076	0,92788	0,191958	0,160686	0,108316	1
ATT:GTA	**0,045659**	0,235102	0,300532	0,14634	1	1
	BIH:MNE	BIH:SLO	BIH:SRB	HRV:FRA	HRV:GRE	HRV:MKD
ATA:ATT	0,264792	0,123942	0,529479	0,987826	**0,012321**	0,939798
ATA:GTA	1	0,**04214**	1	0,858059	0,384771	0,48765
ATT:GTA	0,48765	0,425972	0,930214	0,434723	0,281856	0,819068
	HRV:MNE	HRV:SLO	HRV:SRB	FRA:GRE	FRA:MKD	FRA:MNE
ATA:ATT	0,281856	0,153513	0,434723	**0,029922**	1	0,359251
ATA:GTA	0,50146	0,434723	0,339023	0,930214	0,434723	0,300532
ATT:GTA	0,106599	0,95362	0,96557	0,143503	0,72605	**0,04214**
	FRA:SLO	FRA:SRB	GRE:MKD	GRE:MNE	GRE:SLO	GRE:SRB
ATA:ATT	0,281856	0,967784	0,085953	0,389286	0,108316	**0,006819**
ATA:GTA	1	0,235102	0,235102	0,211144	0,64873	0,136272
ATT:GTA	0,329499	0,434723	1	0,469853	0,389286	0,930214
	MKD:MNE	MKD:SLO	MKD:SRB	MNE:SLO	MNE:SRB	SLO:SRB
ATA:ATT	0,356266	0,384771	1	0,987826	0,136272	0,060527
ATA:GTA	1	0,3067	1	0,226672	1	0,108316
ATT:GTA	1	1	1	0,108316	0,264792	1

Values less than 0.05 are in bold and represent a statistically significant association between variables. The rows represent comparisons between the three different combinations. ALB: Albania; BIH: Bosnia and Herzegovina; FRA: France; GRE: Greece; HRV: Croatia; MKD: North Macedonia; MNE: Montenegro; SLO: Slovenia; SRB: Serbia.

Table S6. List of 40 alternative reference sequences belonging to multiple accessions. The first accession in each row is the label of the corresponding node in Figures 5 and 6.

No. of Accessions	Accession
73	HRV-101, HRV-102, SLO-105S, HRV-105, ALB-10A, HRV-110, FRA-11F, SRB-11, SLO-123S, HRV-123, SLO-125S, HRV-133, SLO-187S, SLO-188S, ALB-18AI, SLO-18T, ALB-19AI, SLO-19T, SLO-1T, MNE-204, MNE-206, MNE-207, MNE-217, ALB-2A, SLO-2T, SLO-2, BIH-305, BIH-307, FRA-30F, BIH-318, SLO-31S, ALB-34AI, FRA-34F, SLO-35S, SLO-37S, ALB-39AI, ALB-3A, MKD-403, MKD-405, ALB-41AI, ALB-43AI, SLO-46S, GRE-4AG, ALB-4A, BIH-510, BIH-515, BIH-519, SLO-57S, SLO-59S, SLO-64S, ALB-6A, SLO-6, HRV-703, HRV-728, HRV-738, HRV-742, HRV-760, SLO-77S, SRB-7, GRE-803, GRE-804, GRE-810, GRE-815, GRE-816, GRE-8AG, SLO-8S, SLO-90S, GRE-9AG, ALB-9A, SLO-9T, SRB-9, SLO-R19, SLO-R3
60	HRV-104, HRV-108, HRV-109, HRV-111, HRV-113, HRV-122, HRV-125, HRV-126, HRV-128, ALB-12A, SLO-132S, SLO-138S, SRB-15, SRB-16, FRA-18AF, SLO-190S, SLO-1, ALB-20AI, MNE-210, MNE-214, SLO-21T, SRB-21, FRA-23F, FRA-24F, SRB-24, SRB-27, SRB-28, SRB-29, BIH-301, BIH-302, BIH-303, BIH-306, BIH-310, BIH-314, BIH-320, FRA-32F, SLO-39S, MKD-402, ALB-42AI, BIH-

	504, BIH-507, BIH-514, BIH-517, SLO-55S, SLO-56S, ALB-5A, BIH-607, SLO-69S, FRA-6F, HRV-715, HRV-717, HRV-739, HRV-747, HRV-755, HRV-756, GRE-7AG, ALB-7A, GRE-801, SLO-91S, SLO-9S
60	REF-5, SLO-102S, HRV-103, SLO-104S, HRV-106, SLO-10T, HRV-112, HRV-117, SLO-11S, SLO-127S, HRV-127, SLO-128S, SLO-129S, SLO-137S, FRA-13F, SRB-13, SLO-14S, FRA-16F, SLO-17T, FRA-19F, FRA-20F, FRA-22F, SLO-22T, SRB-22, SLO-27S, FRA-28F, GRE-2AG, SLO-30S, BIH-315, FRA-33F, SLO-40S, SLO-49S, SLO-4S, BIH-511, GRE-5AG, BIH-605, SLO-67S, HRV-701, HRV-704, HRV-705, HRV-708, SLO-70S, HRV-711, HRV-712, HRV-725, HRV-731, HRV-740, HRV-746, HRV-751, SLO-75S, SLO-78S, SLO-79S, FRA-7F, SLO-80S, GRE-814, SLO-84S, SLO-86S, SRB-8, SLO-97S, SLO-R17
18	FRA-11AF, SLO-186S, SLO-189S, ALB-19A, SRB-19, ALB-1A, MNE-203, SRB-23, ALB-2AI, BIH-311, FRA-31F, SLO-43S, BIH-526, GRE-6AG, HRV-732, GRE-808, FRA-8AF, SLO-94S
16	SLO-113S, SLO-14T, MNE-208, SLO-23S, SLO-23T, SLO-29S, BIH-312, ALB-35AI, SLO-3T, SLO-42S, BIH-518, BIH-520, SLO-73S, HRV-745, GRE-802, GRE-811
13	SLO-108S, HRV-121, SRB-18, MNE-212, SLO-25S, SRB-26, SLO-44S, BIH-516, BIH-602, BIH-611, HRV-714, HRV-719, SLO-93S
7	SLO-103S, GRE-1AG, SRB-20, SLO-68S, HRV-727, HRV-737, SLO-89S
7	REF-2, BIH-317, ALB-37AI, SLO-4, SLO-74S, GRE-813, SLO-83S
7	ALB-33AI, SLO-33S, SLO-3, SLO-47S, HRV-709, HRV-710, HRV-723
6	ALB-11AI, FRA-19AF, SLO-34S, SLO-54S, HRV-706, HRV-729
5	HRV-129, BIH-308, BIH-523, SLO-6T, FRA-9F
4	SRB-12, ALB-17A, BIH-524, ALB-8A
4	SLO-32S, SLO-5T, GRE-806, SLO-81S
4	REF-3, REF-4, SLO-41S, SLO-58S
4	SLO-15T, SLO-7T, GRE-805, SLO-82S
4	HRV-114, ALB-15A, ALB-20A, HRV-722
3	SLO-121S, ALB-38AI, HRV-749
3	SLO-66S, HRV-716, HRV-720
3	HRV-132, SRB-14, MKD-401
3	ALB-16Ai, MNE-215, BIH-304
3	HRV-131, SRB-34, BIH-610
3	ALB-32AI, HRV-735, ALB-9AI
3	SLO-116S, BIH-603, HRV-724
2	HRV-119, BIH-501
2	SRB-10, SRB-30
2	ALB-44AI, HRV-741
2	SLO-19S, BIH-522
2	HRV-734, GRE-807
2	SLO-100S, SLO-101S
2	HRV-118, BIH-609
2	MNE-216, BIH-313
2	BIH-508, HRV-733
2	BIH-509, HRV-702
2	BIH-502, HRV-718
2	MNE-218, SLO-51S
2	SRB-25, MKD-404
2	SLO-16T, SLO-20T
2	SLO-110S, SLO-2S
2	MNE-211, BIH-513
2	SRB-33, GRE-812

Table S7. List of 62 accessions with unique chloroplast genome sequences.

REF-1	MNE-213	HRV-721	HRV-753
REF-6	MNE-202	SLO-72S	HRV-713
GRE-10AG	HRV-116	HRV-730	BIH-608
HRV-115	ALB-21AI	HRV-743	BIH-606
HRV-107	BIH-319	HRV-744	FRA-10F
ALB-10AI	SLO-28S	HRV-754	FRA-14AF
HRV-124	SRB-32	GRE-809	SLO-13T
ALB-11A	GRE-3AG	SLO-130S	SLO-4T
ALB-13A	FRA-3F	SRB-31	SLO-11T
SLO-13S	SLO-45S	BIH-506	SLO-92S
SLO-12T	SLO-21S	BIH-316	SLO-87S
ALB-17AI	BIH-512	BIH-503	SLO-85S
SRB-17	BIH-521	HRV-130	BIH-525
FRA-18F	BIH-601	MNE-209	BIH-309
SLO-18S	BIH-604	HRV-726	
MNE-201	HRV-707	MKD-406	

Table S8. List of probes for seven CpDNA sites analyzed by the target sequencing approach.

Locus	SNP Position	Probes
SNP_NG_C_003	7065	CTTCGACATCATTACATCATTATTTGAGTTGAATAAAGTT<br>TTACAAACAATAAAAAAAACCGCATTTGATCCTTATAAAT<br>AAGAGAGATAAATCCATGTTTCGTTTTGAACTGAACCAAC
SNP_NG_AS_001	33406	CTAGTAATCTTTTCGTTATTGACAAATCGATTGATAATCC<br>ATTTTTCAATGAAAAAAGTATTTTTTTTCTTTACTCTTTT<br>CCTTTCCTTAGACTTTATACTTACAGATCCTGATATGAAT
SNP_NG_D_003	73765	TGAGTCGAAACCAAAGCTCTGATTTTCTGTTGAGTAATAG<br>TTCGAGTAAGTCTTGAATGGGCTCCTCGAAAGCTTGATGT<br>AAATAAACGAATTTTTGTTCTACGTCTACGAGCTATATAT
SNP_NG_C_001	75398	AGTTAAAAAATACTATGATGGCTCCGTTACGTTATATATT<br>TATTTCCTCTATGATTCAGCAATCCCAAAGTTTCTTTTTG<br>ATCTGATCAAATAAAATAAGAACCAAATAAGATTATTTTT
SNP_NG_C_002	123664	TACACCCTATACATGTATCATAAATCTTTACTGAATGTGA<br>CATTGGATCTATAAATTTTTAAACGTCATAAATTTTCGAT<br>CTAGTCAATTTTTAAATGAATCATATATCTAAACACTAGA
SNP_NG_NVD_001	123690	TGTGACATTGGATCTATAAATTTTTAAACGTCATAAATTT<br>TCGATCTAGTAAATTTTTAAATGAATCATATATCTAAACA<br>CTAGATGAATCAATGACTTA
SNP_NG_D_001	128420	CAATTCAAAAGTGGGAGAAGGCTTTTTACTAATAAATCGA<br>TATTCAAAATCATTCAATTCGGGATCCCTCGCTCTATCAA<br>AGCATCGGATTTCTAAATTCTCATACGGCCCTCCCGGAAT

Table S9. Comparison of detected variants between the target and shotgun approaches at seven polymorphic loci.

SNV Position	Number of Samples with Detected SNV		Mismatched Samples
	Targeted Approach	Shotgun Approach	
7065	133	148	ALB-38AI, ALB-42AI, GRE-7AG, SLO-100S, SLO-108S, SLO-121S, SLO-132S, SLO-138S, SLO-190S, SLO-21T, SLO-69S, SLO-91S, SLO-93S
33406	3	0	ALB-38AI, HRV-749, SLO-121S
73765	193	193	/
75398	136	136	/
123664	150	148	BIH-309, BIH-519
123690	192	193	BIH-309
128420	192	193	ALB-2AI
